# In patients with advanced ovarian cancer, primary suboptimal surgery has better survival outcome than interval suboptimal surgery

**DOI:** 10.4274/jtgga.galenos.2018.2018.0015

**Published:** 2019-02-26

**Authors:** Alpaslan Kaban, Samet Topuz, Pınar Saip, Hamdullah Sözen, Yavuz Salihoğlu

**Affiliations:** 1 Clinic of Gynecologic Oncology, İstanbul Training and Research Hospital, İstanbul, Turkey; 2 Department of Gynecologic Oncology, İstanbul University İstanbul School of Medicine, İstanbul, Turkey

**Keywords:** Primary surgery, neoadjuvant chemotherapy, cytoreductive surgery, survival

## Abstract

**Objective:**

It is known that optimal or complete cytoreduction is the most important factor in patients with advanced ovarian cancer. The aim of this study was to examine the results of patients who did not undergo optimal cytoreduction and to examine subgroup analysis based on neoadjuvant chemotherapy (NAC).

**Material and Methods:**

Patients with advanced ovarian cancer and suboptimal surgery were retrospectively reviewed.

**Results:**

A total of 99 patients with a median age of 59.0 years (range, 22-87 years) were studied. The median follow-up time was 39±32.7 months, 81 patients (81.8%) died and 18 patients (18.2%) were alive. The five-year survival rate was 27.6%. Of the patients, 37 (37.4%) were underwent surgery after NAC, 62 (62.3%) were primary. More patients with NAC died within 3 years compared with those without NAC (83.9% vs 56.0%) (p=0.015). Patients with NAC had less tumor spread (presence of visible tumor in the upper abdomen during surgery) (29.7% vs 72.6%; p<0.001) and had less overall survival times when compared with patients who underwent primary surgery [median 22.3±1.2; 95% CI: (19.9-24.7) vs (37.5±11.2); 95% CI: (15.4-59.5) months; log rank test p=0.055]. The relationship between overall survival and factors such as age, NAC, presence of metastasis in the upper abdomen, and tumor histology (serous vs. non-serous) were analyzed using univariate cox regression analysis. Of these factors, only NAC was close to significant, but it did not reach significance (p=0.055).

**Conclusion:**

NAC reduces tumor burden before surgery in advanced ovarian cancer. The prognosis of patients who are not eligible for optimal surgery despite NAC is worse than in patients who do not receive NAC.

## Introduction

Epithelial ovarian carcinoma has the highest mortality among gynecologic cancers (1). The 5-year survival rate is around 30% (2). An important reason for this poor prognosis is that most patients’ disease is diagnosed at advanced stages (3). The standard approach in the treatment of patients with advanced ovarian cancer is debulking surgery for optimal or complete cytoreduction, followed by adjuvant chemotherapy with paclitaxel and carboplatin (4,5,6,7,8,9). The goal is to achieve optimal cytoreduction (less than 1 cm of residual disease), but some patients cannot undergo optimal cytoreduction due to medical comorbidities, experience of the surgeon, intraoperative problems, and disseminated invasive tumor, especially.

Clinical reports provided differing figures about achievable patient rates for optimal debulking surgery. In a large study (1325 patients), the optimal debulking rate was reported as 65% for primary surgery and 74% for surgery after neoadjuvant chemotherapy (NAC) (10). In a randomized controlled trial, Vergote et al. (11) reported their optimal operation rate as 41% in patients who underwent primary surgery and 80% in surgery after NAC. In patients who are not eligible for optimal surgery, administering NAC before surgery is considered as an alternative treatment approach (11,12,13). After NAC, surgical morbidity and postoperative mortality rates are lower and optimal cytoreduction is more likely (11,14,15,16). However, 10-20% of patients who have undergone surgery even after NAC cannot undergo optimal cytoreduction (11,17,18). 

In this study, we reviewed patients with advanced-stage ovarian cancer who could not undergo optimal surgery (residuel tumor >1 cm). We calculated the survival times of these patients and analyzed the relationship between survival duration and age, presence of tumors in the upper abdomen, NAC, tumor histology, and we performed subgroup analysis based on NAC.

## Material and Methods

This retrospective study included 99 patients who underwent suboptimal surgery for advanced stage (International Federation of Gynecology and Obstetrics stage IIIC-IV) epithelial origin ovarian cancer at same center between 2002 and 2013. Patients who underwent suboptimal cytoreduction according to the operation report were selected. The age of the patients, whether NAC was taken, and tumor histology were recorded. According to the operation reports, the presence of visible tumor in the superior part of the liver and diaphragmatic serosa during laparatomy, whether lymphadenectomy was performed, and the number of lymph nodes removed were recorded. The association of these factors with survival was analyzed using univariate Cox analysis.

### Patients

Patients were initially evaluated for gynecologic examination, tumor markers (CA125 and CA19.9) and imaging studies (mostly magnetic resonance imaging). Positron emission tomography examinations were performed in patients as required, and computed tomography was performed in addition to pulmonary evaluation. According to these evaluations, primary debulking surgery was planned for patients who were predicted as being eligeble for optimal surgery. Patients not eligible for primary optimal debulking surgery were deferred for interval debulking surgery after NAC. The current co-morbidity of the patient and the spread of the disease especially (such as liver parenchymal involvement, lung metastasis) was taken into consideration while choosing surgery or NAC. NAC was given after pathologic confirmation of the disease. The standard treatment protocol was 3-6 cycles of paclitaxel 175 mg/m2 and carboplatin (area under curve: 5-6) for 3 weeks. Patients underwent cytoreductive surgery after NAC. At the end of the operation, presence of residual tumor greater than 1 cm was accepted as suboptimal operation.

All patients received adjuvant chemotherapy after surgery. The total (before and after surgery) dose was planned to be 6 to 9 cures. Overall survival (OS) was defined as the time from the first treatment until death or last visit.

Permission of the local ethics committee was not sought because this study was planned as a retrospective review. However, all patients gave informed consent, which allowed our center to use their clinical data for scientific trials.

### Statistical analysis

The Statistical Package for the Social Sciences for Windows version 21 (IBM Corporation, NY: USA, 2012) was used to perform all analyses. Univariate Cox regression analysis was used to investigate the survival- related criteria. Survival distributions were estimated using the Kaplan–Meier analysis. Statistical significance was determined using the log-rank test. P values less than 0.05 were considered significant.

## Results

The study consisted of a total of 99 patients with a median age of 59.0 years (range, 22-87 years). The clinicopathologic features of the patients are presented in Table 1. 

The median follow-up time was 39±32.7 months. Eighty-one patients (81.8%) died and 18 patients (18.2%) were alive. The five-year survival rate was 27.6%. Of the patients, 37 (37.4%) underwent surgery after NAC, and 62 (62.3%) were primary. The comparative analysis of patients who underwent surgery after NAC and primary surgery is shown Table 2. 

**Figure 1:**
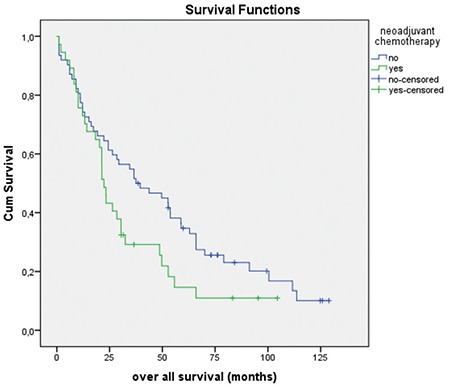
Overall survival graph of patients receiving and not receiving neoadjuvant chemotherapy

Patients with NAC had more deaths within 3 years compared with those without NAC (83.9% vs 56.0%) (p=0.015) (Table 4). Patients with NAC had less tumor spread (presence of visible tumor in the upper abdomen during surgery) (29.7% vs 72.6%; p<0.001) and had less overall survival times when compared with patients who underwent primary surgery [median 22.3±1.2; 95% CI: (19.9-24.7) vs (37.5±11.2); 95% CI: (15.4-59.5) months; log-rank test p=0.055] (Figure 1). The relationship between OS and factors such as age, NAC, presence of metastasis in the upper abdomen, and tumor histology (serous vs non-serous) was analyzed using univariate Cox regression analysis. Of these factors, only NAC was close to significant, but did not reach significance (p=0.055) (Table 3).

More patients with NAC died within 3 years compared with those without NAC (83.9% vs 56.0%) (p=0.015). The distribution of deaths based on the first 3 years and after is presented in Table 4.

## Discussion

In this study, we evaluated survival outcomes of patients with advanced-stage ovarian cancer who could not undergo optimal cytoreduction. Optimal surgery is the most important prognostic factor in survival. Therefore, a good prognosis cannot be expected in these patients. In the study, 81 patients (81.8%) died and only 18 patients (18.2%) were alive during a median follow-up of 39 months. The median OS time was 29 months. Some of these patients had received NAC and the prognosis of these patients was worse (22 vs 37 months, median) (Table 3). The death ratio was found higher, especially within 3 years, in the NAC group (Table 4). In fact, the tumor burden during surgery was less in patients who received NAC but this was not positively reflected in survival (presence of residual tumor in the upper abdomen 29.7% vs 72.6%, p<0.001).

In the literature, there are studies comparing patients receiving NAC and patients not receiving NAC. Some survival of patients who received NAC. According to the recently published Danish retrospective cohort study in which 1734 patients were evaluated, survival was found to be lower than in the primary surgery group in patients with stage IIIC ovarian cancer who underwent surgery after NAC (29.4 months, 33.7 months; p=0.057) (10). In their study, patients who had no residual tumor at the end of surgery were also compared and survival was found to be significantly lower in the NAC group (36.7 and 55.5 months; p=0.002). In addition, long-term survival (more than two years) was significantly lower in their study. According to the authors, treatment with NAC may impair long-term survival. In the study of the European Organization for Research and Treatment of Cancer, 55971 patients with stage III ovarian cancer had better survival in the primary surgery group compared with the NAC group (19). In a Surveillance, Epidemiology and End-Results data study in which 6844 patients were evaluated, NAC increased the risk of death by 16% for patients with stage III disease at two years (20). Rosen et al. (21) reported that 7-year survival was significantly better in the primary surgery group than in the NAC group (8.6% vs 41%; p<0.0001). In a meta-analysis involving 835 patients, NAC in lieu of primary cytoreduction was associated with inferior OS compared with initial surgery (22). In another study, Ren et al. (23) reported worse survival in the neoadjuvant group in a study involving 408 patients.

There are different opinions as to why the survival of patients with ovarian cancer who receive NAC is worse than in patients who undergo surgery without NAC. However, these groups are not similar enough to compare. Primary debulking surgery is planned for patients who have been predicted as being eligible for optimal surgery. Patients not eligible for primary optimal debulking surgery are deferred to interval debulking surgery after NAC. This indicates that patients were selected for surgery upfront if they were deemed "debulkable", whereas those who received NAC appeared not to be candidates for complete debulking. One cannot draw any conclusions about the difference in outcome because these two cohorts have different disease burden and biology, which is expected to be worse in patients who are not candidates for primary surgery. An another proposed idea is that NAC has a deceptive effect on intraoperative evaluation. Due to the effect of chemotherapy on the tissue, the tumoral area may be missed, and difficulty of resection of potentially resectable tumor tissues may have a negative effect (24,25). Another sugestion is that NAC induces the emergence of chemotherapy-resistant tumor cells in stem cell colonies over time. There are reports that NAC increases the risk of platinum resistance over time (26,27,28). It is important at this point to consider platinum resistance in the selection of patients for NAC. Currently, platinum resistance is not tested in patient selection and there is no such recommendation in guidelines. It may be useful to develop and apply *in vivo* chemosensitivity testing, which may show primary platinum resistance (29). Another hypothesis is that delayed debulking surgery may also adversely affect survival (30).

The contribution of NAC to survival is not clear in the literature, currently. According to the results of randomized controlled trials, the general consensus suggests a similar survival rate between primary surgery and interval surgery after NAC (11,31,32,33,34). NAC improves the feasibility of optimal surgery by decreasing tumor spread (16). However, optimal surgery may not be possible despite NAC. In the study of Fagö-Olsen et al. (10) 180 of 515 patients who received NAC did not undergo surgery (predominant reason was that the tumor was considered to be unresectable) and the median OS of these patients was 14.3 months. The authors reported that it was controversial as to whether these patients recovered from unnecessary surgery or that they were deprived of the possible advantage of surgery. 

The present study has some limitations such as the low number of patients and its retrospective design. In addition, all patients were those with advanced ovarian cancer who underwent suboptimal surgery, but residual tumor burdens may be different.

This study evaluated a group of patients with advanced ovarian cancer who underwent surgery for optimal cytoreductive surgery but underwent suboptimal surgery. Even with NAC, some patients may not be feasible for optimal surgery. The prognosis of these patients is poor. It is controversial as to why chemotherapy does not contribute to survival despite tumor burden reduction. In vitro studies on the relationship between chemotherapeutic agents and tumor cells may be informative.

## Figures and Tables

**Table 1:**
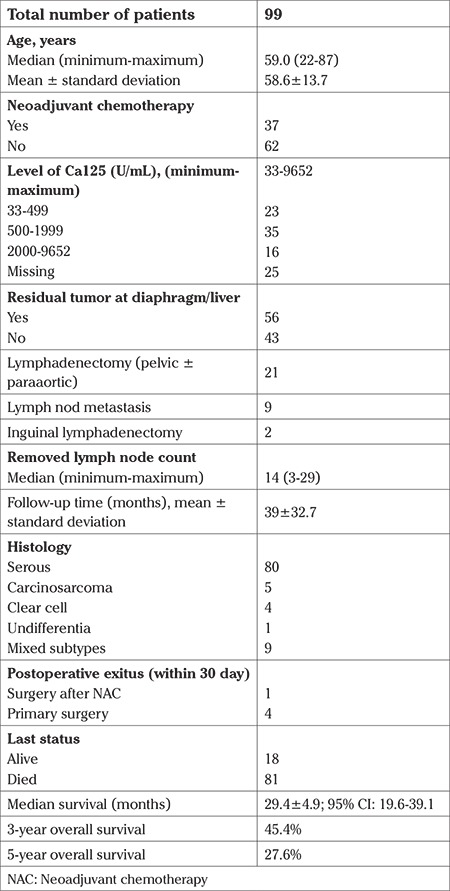
Clinical features of patients

**Table 2:**
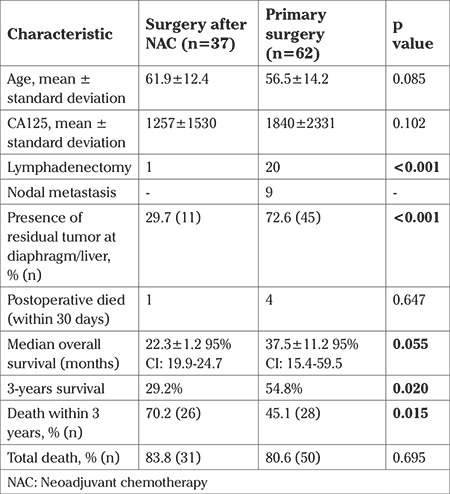
Comparative analysis of patients with operated after NAC and primary surgery

**Table 3:**
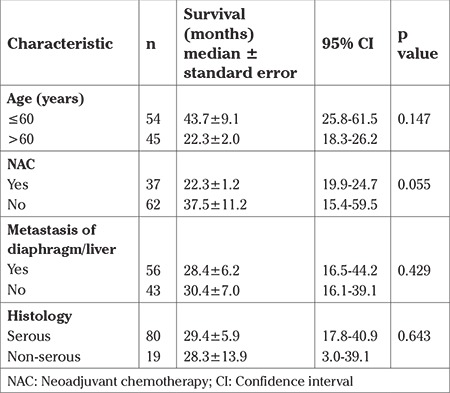
Analysis of survival related factors by univariate Cox analysis

**Table 4:**
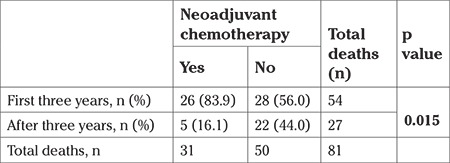
Distribution of 81 deaths in the first three years and after
